# Peer Ambassador Perspectives in a Culturally Tailored Self-Management Intervention for African Americans with Type 2 Diabetes: A Qualitative Study

**DOI:** 10.3390/pharmacy12030075

**Published:** 2024-05-08

**Authors:** Meng-Jung Wen, Ejura Y. Salihu, Choua Yang, Martha Maurer, Olayinka O. Shiyanbola

**Affiliations:** 1 Division of Social and Administrative Sciences in Pharmacy, School of Pharmacy, University of Wisconsin-Madison, Madison, WI 53705, USA; mwen8@wisc.edu (M.-J.W.); salihu@wisc.edu (E.Y.S.); 2 School of Pharmacy, University of Wisconsin-Madison, Madison, WI 53705, USA; cyang453@wisc.edu; 3 Sonderegger Research Center for Improved Medication Outcomes, School of Pharmacy, University of Wisconsin-Madison, Madison, WI 53705, USA; mamaurer@wisc.edu

**Keywords:** African Americans, diabetes, peer support, self-management, stakeholder engagement

## Abstract

Objective: Diabetes disproportionately affects African Americans, leading to higher morbidity and mortality. This study explores the experiences of African American adults who successfully self-manage their type 2 diabetes (called Peer Ambassadors) and provided phone-based peer support in a 6-month culturally tailored diabetes self-management program for African Americans guided by the information–motivation–behavioral skills model. Design: A group discussion using a semi-structured discussion guide was conducted. Qualitative content analysis was used to identify the facilitators and barriers to completing the role of a Peer Ambassador and to develop strategies for overcoming possible challenges in the future. Setting: Key informant discussions were conducted in a community location to gain insights into Ambassadors’ motivations and challenges in delivering peer support. Participants: Three Peer Ambassadors completed ethics training and peer mentor training and received a phone call guide before providing support to their peers. Results: There were four core themes related to Peer Ambassador experiences: (1) Motivation to be a Peer Ambassador, (2) program elements that supported Peer Ambassador role, (3) key elements of achieving engagement, and (4) challenges related to being a Peer Ambassador. Conclusions: This study showed Peer Ambassadors in a culturally tailored peer supported self-management program found fulfillment in sharing experiences and supporting peers. They highly valued educational group sessions for knowledge updates and sustaining their health-related goals, suggesting the potential benefits of recognizing milestones or providing advanced training for future program sustainability. Findings suggest the importance of recruiting motivated patients and providing effective facilitation for peer support roles, including addressing barriers such as time commitment and lack of socialization opportunities.

## 1. Introduction

Diabetes is a chronic disease that affects 37.3 million people in the United States and among this population, 95% have type 2 diabetes [1]. Compared to non-Hispanic white adults, African American adults are 60% more likely to be diagnosed with diabetes and are more likely to experience diabetes-related kidney disease and amputations compared to white adults, leading to increased hospitalizations and death [2,3,4]. Addressing diabetes disparities requires reducing hemoglobin A1C. Though elevated A1C is driven by varying factors, studies show that medication nonadherence is one of the strongest predictors [5,6,7]. African American adults have a lower rate of adherence to diabetes medicines, contributing to elevated A1C and, if left unaddressed and uncontrolled, could lead to increased mortality and morbidity [8,9].

Prior studies have shown that the differences in medication adherence could be attributed to beliefs concerning diabetes and its medications [10], as well as low self-efficacy and patient–provider interactions affected by provider distrust [11]. Also, social determinants of health such as food insecurity, transportation, and access to healthcare services were found to be common barriers to medication adherence and diabetes self-management among African American patients [12,13,14,15]. Despite the role of system-based interventions to address these social needs, beliefs, self-efficacy, and communication between patients and providers can be altered in a relatively short period, making them potential targets for effective tailored interventions [7].

Managing diabetes is physically and emotionally challenging for patients and providing peer support via peer mentors can offer a practical and flexible method of providing personalized support, beyond that of a healthcare professional. In previous studies, race-congruent peer ambassadors played vital roles in addressing the health beliefs of their support persons and addressing adherence to medication use [16,17,18]. In diabetes self-management interventions, peer mentors can draw from their own experiences with the disease to effectively assist others in managing their condition [19,20]. By creating a nonhierarchical and reciprocal relationship based on shared experiences and cultural beliefs, this type of peer support can significantly engage patients and align with the social dynamics of their community [21]. Research has demonstrated that such peer-led interventions can provide culturally appropriate support and increase medication adherence beyond what clinicians can achieve through clinic visits alone [22,23]. Peer support also enhances self-advocacy in communicating with healthcare providers [24]. Thus, for African American adults who may have beliefs about diabetes and mistrust towards medicines due to cultural and historical factors, race-congruent peer support is particularly important and effective [25].

The parent study focuses on a 6-month diabetes self-management program that was culturally tailored to African Americans (called Peers EXCEL) and aims to address medication beliefs and medication nonadherence and may improve diabetes outcomes [26]. We developed Peers EXCEL using the information–motivation–behavioral skills model to incorporate education and motivation for positive attitudes towards adherence via peer support and increased self-management skills via education on promoting provider–patient communication and developing skills to increase self-efficacy [27,28]. Peers EXCEL engages healthcare professionals in providing tailored education and support as well as race-congruent peer support from other African Americans with diabetes. Despite the availability of several diabetes self-management programs, prior programs do not adequately address the psychosocial barriers that prevent successful diabetes self-management in African Americans due to insufficient attention to predisposed health beliefs, related to medication adherence, and provider distrust [29]. Peers EXCEL aimed to address these issues by providing culturally relevant education and utilizing peer support to enhance motivation for self-management, medication adherence, and provider trust.

African Americans with type 2 diabetes who successfully self-managed their diabetes, called Peer Ambassadors, were paired up with participants who needed further support, called Peer Buddies. Some advantages of incorporating peer support when working with marginalized populations is that the program activities and education sessions become relevant to their lifestyles and enhance credibility of the program within the community [21]. Due to Peer Ambassadors having relatable backgrounds and similar chronic disease experiences as the Peer Buddies, they have greater insight into the psychosocial barriers that may influence their ability to self-manage, contributing to further conversations on how to improve medication adherence and glycemic control for African Americans with diabetes [13]. Exploring feedback given by Peer Ambassadors and learning about their perspective are important ways to help improve future diabetes self-management programs for the African American community. Understanding their stories, motivations, and challenges will enhance knowledge for program effectiveness and ways to improve engagement between Peer Ambassadors and Peer Buddies.

There is limited literature that incorporates peer leaders’ perspectives in peer support interventions [30]. This paper aims to provide insight into peer leaders’ experiences with providing peer support to African Americans with diabetes to obtain a unique understanding about peer leaders’ motivations and challenges, what they enjoyed and disliked, and what was helpful and not helpful.

## 2. Materials and Methods

This research used qualitative content analysis to capture Peer Ambassadors’ insights into their experiences and role in Peers EXCEL [31]. Three Ambassadors were the key individuals who provided peer support. Hence, key informant discussions were conducted initially at the end of the 8-week group sessions in August 2021 and before the end of the overall 6-month program in November 2021. We gathered feedback on their participation as Peer Ambassadors and received suggestions for future improvement of the program based on their common experiences [32]. This study was exempted by the Principal Investigator University Health Sciences Institutional Review Board (STUDY ID: 2020–1061) since the study procedure only included program evaluation.

### 2.1. Participants and Program

Though this study aims to explore Peer Ambassadors’ insights into their experiences, and roles, we provide a brief overview of the program to give context to the Peer Ambassadors’ experiences.

#### 2.1.1. Overview of Peers EXCEL

Peers EXCEL is a six-month culturally tailored diabetes self-management program in Milwaukee, a metropolitan city of about 600,000 in Wisconsin, USA. The Peers EXCEL program is a result of the integration of a prior program, called Peers LEAD [33] with particular focus on medication adherence and addressing beliefs, into a widely disseminated evidence-based program—Healthy Living with Diabetes (HLWD). The details regarding program design, participant recruitment, and intervention outcomes of the Peers EXCEL program are published elsewhere [26,34,35]. 

Peer Ambassadors are African Americans with type 2 diabetes who report being adherent to diabetes medication. Peer Buddies with type 2 diabetes who reported being nonadherent to their diabetes medications were paired with Peer Ambassadors and interacted together during the program group education sessions and phone calls [33]. 

Healthy Living with Diabetes (HLWD) is a community-based evidence-based program that offers tools to enhance understanding of what it means to have diabetes, including helping participants to build the confidence to manage diabetes and maintain an active and fulfilling life [29]. HLWD is proven to reduce emergency department visits by 53%, improve A1C levels, enhance regular treatment and diabetes education, and improve self-rated health, communication with physicians, and self-efficacy among the general population [36,37]. Despite its success, similar to other diabetes self-management programs, African Americans are less likely to participate in HLWD and have better diabetes outcomes [36,37,38].

The content of group sessions in the Peers EXCEL program was culturally adapted for African Americans by adding components of Peers LEAD (culturally influenced diabetes and medication belief information, behavioral skill development, and one-on-one race-congruent peer support) to overcome key barriers to African Americans’ medication adherence that HLWD alone does not sufficiently address [29,39,40,41]. Peer Buddies attended the first two group sessions led by healthcare professionals to discuss beliefs about diabetes, healthcare mistrust/discrimination, and provider communication, followed by six weeks of diabetes self-management education group sessions led by certified HLWD facilitators. After completing the group sessions, five weekly phone calls and subsequent monthly calls to Peer Buddies occurred to provide further support for diabetes management.

#### 2.1.2. Recruitment of Peer Ambassadors

Community stakeholders from previous partnerships helped with approaching and introducing potential candidates for Peer Ambassadors [42,43,44]. Purposive sampling was used to recruit participants who: (1) scored above 11 on the self-reported Adherence to Refills and Medications Scale for Diabetes (ARMS-D) scale, indicating adherence to their diabetes medication, (2) were willing to provide support to a Peer Buddy and track conversation in phone calls, and (3) were willing to participate in all Peer Ambassador training sessions and meetings. We also invited Peer Ambassadors from our prior program to participate in the new program.

#### 2.1.3. Peer Ambassadors’ Training and Role before 8-Week Intervention

Prior to the program, Peer Ambassadors had to complete the human subject ethics training and sign an investigator responsibility form approved by the University Institutional Review Board. A one-hour brief orientation virtual meeting via Zoom hosted by the PI introduced the program components, intervention timeline and contents, time commitment and payment, and the overview of peer support phone calls. The training of Peer Ambassadors was co-coordinated by the PI and a consultant from the Wisconsin Network for Research Support (WINRS), an organization that facilitates recruitment and patient and community engagement for research [45,46]. The two training meetings were six hours in total, which prepared Peer Ambassadors for their roles and responsibilities in the program. The components of the training comprised a review of program objectives and the use of intervention guides and manuals. The research team members explained thoroughly the guides for peer support phone calls and conducted role-play as one of the critical approaches to let them familiarize themselves with the use of guides as well as their role as Peer Ambassadors [42,43]. Table 1 demonstrates the activities that Peer Ambassadors participated in along with the timeline of implementing the Peers EXCEL program.

#### 2.1.4. Peer Ambassadors’ Role during and after the 8-Week Intervention

During the 8-week intervention, Peer Ambassadors attended the group sessions with Peer Buddies to learn diabetes self-management topics and engage in the discussions. They also conducted weekly phone calls for the first two weeks of the sessions to reinforce the educational content and provide introductions to their buddies. Two weeks after the eight-week sessions, Peer Ambassadors started weekly follow-up phone calls to support their buddies using the standardized manual and guide. Group session topics, content, and discussion were emphasized again during their conversation. As well, they discussed their goals, any barriers to meeting the goals, and strategies to solve problems related to diabetes self-management. During the peer support phone calls, Peer Ambassadors also documented the content, questions, and concerns raised in the conversations. From weeks 15–24, Peer Ambassadors further reviewed the achieved goals with their Peer Buddies and sought for other solutions to reset the goals if possible. Peer Buddies were able to call their ambassadors during these periods at their discretion.

### 2.2. Data Collection

We used convenience sampling to recruit Peer Ambassadors. The eligibility criterion was Peer Ambassadors who participated in the six-month Peers EXCEL program. The PI, an African American researcher with extensive training in qualitative methodology and experience facilitating group discussions, moderated the group discussion. One scientist who is also trained in qualitative research methodology and has performed several qualitative studies in a similar field observed and took notes. One Peer Ambassador was unable to participate in the discussion due to scheduling conflicts, so a one-on-one discussion was conducted by the PI instead. Using appropriate probing questions, the facilitator elicited robust comments and ensured that all participants were equally engaged in the discussion throughout the discussion [32]. All discussions were conducted virtually via Zoom during the COVID-19 pandemic. Each discussion lasting 90 min and one-on-one interview lasting 60 min was audio-recorded. A professional transcriptionist transcribed the transcripts verbatim. Afterward, the transcripts were reviewed by a research assistant to ensure accuracy. The discussion guide (Table 2), developed by the PI, a researcher and WINRS, includes questions aimed at eliciting feedback on different aspects of the program, e.g., the group educational sessions, phone calls with Peer Buddies, and support from the research team. The participants were also asked to share advice that could aid the recruitment and retention of Peer Ambassadors in future iterations of the program.

### 2.3. Data Analysis

This study used an inductive qualitative content analysis approach to investigate the experiences, feedback, and advice related to being a Peer Ambassador [31]. A naturalistic paradigm was applied in this study to explore the perceptions and experiences of Peer Ambassadors who completed the Peers EXCEL program. Two researchers, trained in qualitative research, analyzed and managed the three qualitative transcripts using NVivo 10 (Lumivero). The researchers independently conducted open coding on one transcript and met to compare their codes before agreeing on a codebook. Then, they independently coded all the transcripts, categorized the codes into categories, and then conducted abstraction by combining their codes into categories. They also reviewed and compared categories focused on the following research questions: (1) What are the motivations for being a Peer Ambassador in Peers EXCEL? (2) What support and training help in adapting to the role and responsibilities of being a Peer Ambassador? (3) What are the elements of building and extending the relationship with Peer Buddies? (4) What are the challenges of being a Peer Ambassador? (5) What could be carried out for future program improvement?

### 2.4. Rigor

Principles from Lincoln and Guba’s work on the trustworthiness of qualitative studies were evaluated [47]. The credibility of the study was established by the researchers who immersed themselves in the qualitative data by reading line-by-line through three transcripts. A secondary review of the findings by an advisory board of qualitative researchers external to the study team was conducted to ensure the credible interpretations of the data. Thick descriptions of the method, data collection, and analysis were provided to aid the transferability of findings to other similar settings. Two investigators independently developed the codebook and analyzed the data. Audit trails of each modification of the procedure of data collection and analysis were conducted to ensure confirmable findings and interpretations. 

## 3. Results

A total of two discussions and one interview with the three Peer Ambassadors who provided peer support in this study were conducted. There were two women and one man with a mean age of 56 and mean duration of diabetes diagnosis was 9 years (SD = 4.6). Qualitative analysis identified Peer Ambassadors’ perceptions and experiences after participating in the Peers EXCEL, including: (1) motivation to be a Peer Ambassador, (2) program elements that supported the Peer Ambassador role, (3) key elements of achieving engagement, and (4) challenges related to being a Peer Ambassador. Table 3 shows the themes, subthemes, and representative quotes.

### 3.1. Motivation to Be a Peer Ambassador

#### 3.1.1. Powerful Opportunities to Positively Impact the African American Community

Peer Ambassadors perceived that the core element of their motivation is to help people in their community. Sharing the same cultural background and experience motivated them to help other people in the community with the same condition.

“I have a lot of people in my family that’s been affected by diabetes and … I’m so glad that there’s a program now that’s in place to give us the knowledge and the resources because I didn’t have that. I’m sure none of my family had a chance to experience something like this, an opportunity like that…this is life changing. And that’s the reason why I would be in the program because it is helping people become better. And it’s our people”—Peer Ambassador

#### 3.1.2. Being in a Prior Similar Situation with Lack of Information and Support

Reflecting on their own situation while newly diagnosed, all Peer Ambassadors expressed an urgent need for information and resources related to diabetes self-management. Therefore, as people who had been through the journey of diabetes, they wanted to change their role to supporting others.

“I just remember when I was diagnosed and not having any support, anyone to talk to, not knowing what to do, how to talk to a doctor, how to talk to a pharmacist, what numbers meant. And when you get someone who’s changed because of the information they received, it’s very motivational. …So primarily, it’s just if I can do anything or help anyone with their numbers or understanding all the food and reading labels and exercise. It’s not that I’m 100% correct or anything like that, but I do know how to take my medication”—Peer Ambassador 2

#### 3.1.3. Paying It Forward by Sharing One’s Experience

Peer Ambassadors in this program not only enjoyed sharing their experience but valued the opportunities of keeping their diabetes-related knowledge updated. They shared their experiences and emotions with Peer Buddies as they were in the same boat as Peers Buddies not long ago. One of the Ambassadors shared that being recognized as a Peer Ambassador means they mastered the skills learned from the program and had the ability to support others.

“I think that’s big time to be able to have a Peer Buddy. It also just kind of gives them recognition that, hey, you went through a program. You get it. You understand. You were helped. And now your testimony can help somebody else. I think that’s powerful”—Peer Ambassador 3

### 3.2. Program Elements That Supported Peer Ambassador Role

#### 3.2.1. Personal Benefits from Attending Sessions with Buddies

To fulfill the Peer Ambassadors’ role and responsibility, the Peers EXCEL program prepared informational and motivational support and resources to build relationships with Peer Buddies. The importance of attending group sessions was emphasized by all Peer Ambassadors since the contents and discussions in group sessions can be naturally integrated into their conversations with Peer Buddies.

“[Attending the group sessions] helps you stay on your game. And it keeps you on top of, and then you get to add to that conversation, your experiences, and let them know that you’re no different. You struggled at it one time. You got to this point. But they get to have a personal relationship with you also”—Peer Ambassador 2

#### 3.2.2. Session Information about Goal Setting and Self-Management Led to Personal Commitment

Peer Ambassadors’ particularly recognized that goal-setting components during group sessions kept all participants motivated to implement what they learned in their daily life.

“I think the main topics of diet, exercise, they were so key, and they were so motivational to the Buddies and us. Just like [Peer Ambassador 3] said, we weren’t even required to make commitments. And we all did just because of that energy level”—Peer Ambassador 2

#### 3.2.3. Resources and Guides Enhanced Role Preparation

Apart from the group sessions, Peer Ambassadors appreciated the guidance of the phone calls with Peer Buddies. Provided by the research team, the guidance included weekly topics and scripts which could be used to facilitate their conversation with Buddies.

“We have an outline of… when to call and just different topics and things that we should be talking to our Peer Buddies about as well, which is very helpful. Not that we have to follow the script, but at least that gives us a guide, like a map that we should be following just to make sure that we’re hitting those certain touch points”—Peer Ambassador 3

### 3.3. Key Elements of Achieving Engagement

#### 3.3.1. A Comfortable and Safe Environment

Making constant efforts to keep all participants motivated was critical throughout the Peers EXCEL program. Peer Ambassadors mentioned the importance of creating a welcoming atmosphere where all participants could comfortably share their concerns without being judged. As well, one Ambassador acknowledged the inclusive culture of the program in that everybody is willing to participate in group discussions to share their own stories. This shows that the participants were engaged and participated in the program.

“I thought they participated well. They told personal stories. It was like a little group, a little family. You know, everybody all participated. …everybody told their story and felt comfortable to tell their story in that environment. That was really important too. So I thought they [Peer Buddies] were engaged”—Peer Ambassador 2

#### 3.3.2. Facilitator Skill in Engaging Participants in Educational Sessions and Enhancing Participants’ Accountability

All Peer Ambassadors agreed that the facilitator’s skills are central to keeping participants engaged with the educational content that Peers EXCEL aimed to deliver. An energetic personality with an interactive style of presentation were frequently mentioned as their favorite type of facilitator. Moreover, they also appreciated the regular check-ins and follow-ups for everyone in the discussions and perceived this strategy as essential to enhancing participants’ accountability and ownership of the goals they want to achieve.

“It was very huge having her [the facilitator in HLWD] come and kind of do her things and doing check-ins and different things like that, keeping people engaged and, holding people accountable but not being too tough on them and giving people goals week to week… they’re able to kind of take ownership in the things that they decide that they want to do. So I thought that was very awesome… just her energy and personality… it was unbelievable”—Peer Ambassador 3

#### 3.3.3. Discussion of Culturally Tailored Elements Aligned with African Americans’ Needs

Giving examples aligned with their culture and daily routine maximized the utilization of self-management knowledge in the group sessions. Participants in this study demonstrated that the content in some of the group sessions did not really match what they need. However, the group of African Americans discussing the real situations based on their daily life enriched the information that could be applied to themselves.

“Having the group of African Americans and talking about their experiences, and then when you go to diet, they’re bringing the food that they like. And it’s something we all like… by having [the facilitator in HLWD], answering questions specifically that we can relate to. Go to a barbecue…Go get the greens, the yams… I may not have gotten that information in another diabetic class, except for we know that we like those things at barbecues”—Peer Ambassador 2

#### 3.3.4. Peer Accountability and Empowerment

Peer empowerment served as one of the most significant goals for Peer Ambassadors to provide peer support. Starting from introducing themselves in the first and second phone calls, Peer Ambassadors continually interacted with their Buddies to ensure they were walking together through the journey. One Peer Ambassador expressed how peer impact is sometimes greater than family’s influence on managing disease.

“When you educate people, and they understand how they should be eating, how serious they should take their medication, how they should be engaged and have accountability partners with the group and people that’s checking on them, people that care about them, you know what I’m saying, that’s not even part of their family but just part of a program, to be a part of something, that is huge”—Peer Ambassador 3

### 3.4. Challenges Related to Being a Peer Ambassador

#### 3.4.1. Hard to Maintain Individual Commitment with Long Session Length and Program Duration

The commitment to engaging in the program was the biggest challenge to the Peer Ambassador role. Despite the informative program, participating in the weekly sessions for six weeks was challenging for one Peer Ambassador. Regarding the one-on-one phone calls, one peer Ambassador was frustrated when they tried several times but could not reach out to Peer Buddies who had less passion for the program.

“I think it got hard for people to maintain that schedule for that length of time. And I always thought that was a problem with that particular program. I think people tried very hard… Some was so dedicated because they were getting so much out of it from [the facilitator in HLWD]”—Peer Ambassador 1

“Sometimes it would take three to five phone calls to get to that one conversation, that when we had that conversation, it was great… I just wanted it to be as important to them, you know, that you think about my time too, that it shouldn’t take me four to five phone calls to get to you. That was my biggest challenge”—Peer Ambassador 2

#### 3.4.2. Easily Distracted in a Virtual Meeting

The other challenge that participants pointed out was that it was hard to stay engaged via virtual interactions. All Peer Ambassadors preferred in-person sessions to have more participation and opportunities to socialize. One Ambassador was also concerned that their Buddies would get distracted with other things and lost motivation to stay in the program.

“Either towards the end or the middle where we get a chance to kind of come together and see each other… I just think that would kind of be motivating. …because a lot of times, when you’re doing so much virtual…, your family, your kids that’s around. So you’re just kind of a little distracted where you’re not in tune as much… if we can do some in person but then do these virtual, I think that’s maybe… good and keep the Peer Buddies motivated”—Peer Ambassador 3

“I just think the virtual stuff is just a killer. But if it’s in person, the program is just a lot smoother, more participation. And you can kind of spread it out, you know what I mean, engage, socialize a little bit. So you can pick your points… and people still get the education they need”—Peer Ambassador 3

## 4. Discussion

This qualitative study investigated the experiences of Peer Ambassadors who participated in the Peers EXCEL program, a culturally tailored diabetes self-management program that incorporated peer support [33]. The findings showed that personal factors as well as program-related factors were critical in ensuring successful implementation of the program. Personal factors that informed the Peer Ambassadors’ decision to enroll and complete the program included the desire to help others in the community who are struggling with diabetes management and personal need to gain additional knowledge on diabetes and diabetes self-management. On the other hand, program-related factors (e.g., the peer-support component of the program, group session facilitators’ personalities, and the culturally tailored instructional materials used) were critical to enhancing the successful implementation of the group sessions. All Peer Ambassadors noted the desire to improve diabetes management in the African American community as a major motivator for enrolling in the program as peer support persons. Closely related to the desire to help their community, Peer Ambassadors reported gaining immense satisfaction from sharing their experiences and supporting their Peer Buddies [43]. 

Apart from the altruistic benefits, Peer Ambassadors appreciated the opportunity to update their understanding of diabetes and diabetes management during the group education sessions [48]. Peer Ambassadors reported that they incorporated the knowledge gained from the group education meetings to improve their health while also helping their Peer Buddies develop self-efficacy for diabetes self-management. This, in turn, improved Peer Ambassadors’ self-efficacy in the role. This finding is corroborated by the parent study research, which explored the experiences of Peer Buddies who participated in the Peers EXCEL program [33]. Similar to the Peer Ambassadors, Peer Buddies stated that the knowledge gained during the group education sessions improved their understanding of diabetes and self-efficacy and empowered them to disregard their previously held negative beliefs about diabetes and diabetes medicines. 

According to Peer Ambassadors, the group session facilitator’s ability to ensure participants felt safe and heard during the session increased engagement. Peer Ambassadors expressed a preference for facilitators who were energetic and confident with an interactive presentation style. Facilitators’ ability to convey the information in the instructional materials in simple, easy-to-understand language and apply culturally tailored examples of diet options were also noted as factors that improved participants’ learning and engagement. These findings have significant implications, indicating that the quality of delivering educational group sessions is highly dependent on the facilitator’s ability to lead an engaging session and the cultural suitability of the program content. This result resonates with previous evidence [33] that diabetes self-management programs that use culturally tailored instructional materials have high recruitment and retention rates as well as better health outcomes such as improved African Americans’ diabetes self-management and positive change in beliefs about diabetes and diabetes medicines [49]. 

Other program-related factors that supported Peer Ambassadors’ participation in the program included the extensive training they received prior to starting the program, access to guides for discussions with their Peer Buddies, and frequent check-ins by the research team. Peer Ambassadors believed that the research team’s hands-on approach to training and ongoing support helped them fulfill their duties to the Peer Buddies effectively. Most Peer Ambassadors stated that frequent check-in calls from the research team provided accountability and an opportunity to take ownership of their role. 

To enhance participant acceptability and program fidelity, researchers should recruit facilitators or group leaders who can build trust with the participants by being culturally aware and nonjudgmental. Previous studies have shown that trust-building is an important component of successful peer-supported interventions [50,51,52]. To improve self-disclosure among African Americans with diabetes and maximize the benefits of diabetes education sessions, it is crucial that researchers provide facilitators with additional training on delivering culturally relevant materials and creating an active learning environment where participants feel safe to ask questions and share their experiences without judgement [52]. 

According to Peer Ambassadors, the barriers to participation were (1) the time commitment required to participate in the program, (2) unequal motivation levels between Peer Ambassadors and their Peer Buddies, and (3) the virtual nature of the group sessions, which provided a very limited opportunity for socializing and building rapport with peers. Despite their unanimous praise of the educational value of the group sessions, Peer Ambassadors felt that the group sessions were too long. It was suggested that future sessions should be shorter to reduce participants’ fatigue. Another major barrier to participation was the unequal motivation levels between Peer Ambassadors and their Peer Buddies. One Peer Ambassador reported that they noticed that some Peer Buddies were not as motivated to engage with their ambassadors and improve their self-management as others. This unequal weight of motivation meant that some Peer Ambassadors could not reach their buddies for a few weeks, and when they did, the conversations were one-sided. In addition, Peer Ambassadors preferred in-person sessions because they felt in-person meetings would offer them the opportunity to be more engaged and socialize, improving their experience in the program. To reduce the attrition of participants in future programs, they suggested sessions should be shorter (less than two hours), and group sessions should be held in person rather than virtually so that participants are able to build rapport, which is a key element of building engagement and trust. Researchers should also consider matching Peer Ambassadors with Buddies of similar communication styles and motivation levels. Efforts should also be made to ensure Buddies have the support they need to connect with their Peer Ambassadors and engage in the program fully. 

Overall, Peer Ambassadors’ feedback on the cultural fit of the instructional materials and other aspects of the program showed that the Peers EXCEL program design and delivery were appropriate and acceptable. An understanding of the personal factors such as the personal need for diabetes self-management knowledge and wanting to help other African Americans living with diabetes and the program-specific factors (facilitators’ presentation style and engaging personality, cultural appropriateness of the program content, and the peer-supported format of program) facilitated the Peers EXCEL program and may provide a helpful framework for designing future peer-supported interventions in African American communities. 

Incorporating the input of Peer Ambassadors on the facilitators and barriers to their participation in the program can help with recruitment strategies and participant retention. Knowledge gained from meetings with the Peer Ambassadors has provided insights into what the community needs and how researchers can better connect and engage with them when conducting similar studies. From the Peer Ambassadors’ perspective, researchers should place great emphasis on recruiting self-motivated patients who are interested in their community’s health for the role of Peer Ambassadors. To enhance program sustainability and maintain high levels of motivation among Peer Ambassadors, future programs should consider establishing several milestones for Ambassadors who aim to advocate for diabetes self-management in their community. One such milestone could involve connecting them to the HLWD structured training, enabling them to acquire the necessary skills to become effective group leaders. Future program implementation should include providing an in-person option for group sessions, and emphasis should be placed on recruiting African American facilitators that are confident and engaging. Considering that Peers EXCEL is an eight-week program, thus requiring several weeks of consistent commitment from participants, efforts should be made to recruit Peer Buddies who self-report motivation and readiness for the program from the outset.

## 5. Strengths and Limitations

The use of discussions to explore Peer Ambassadors’ experiences in the Peers EXCEL program is a key strength of this study because it allowed participants to discuss perceived benefits of the program and make recommendations for potential program improvements based on their common experiences. The group dynamics allowed the participants to share, disagree, and clarify their views on the Peers EXCEL program, which is usually less obtainable in one-to-one interviews and other qualitative techniques [53].

It is possible that these results are limited to a small number of peer leaders who share similar characteristics because only three Peer Ambassadors were involved in the program and do not reflect the breadth of other peer experiences prevalent in Black/African American populations. This study was conducted in a Midwestern urban metropolitan city with a large Black/African American population, suggesting that experiences and motivations for being peer leaders could differ in other geographic regions. Similar studies should be cautious that the sociocultural backgrounds and healthcare infrastructure may vary when the results are applied to other settings. However, findings from this study have established a stepping stone for conducting trials on a larger scale. With more participants and peer leaders included, the effectiveness of Peers EXCEL can be assessed and diverse experiences of providing peer support from peer leaders’ perspectives can be gathered.

Due to the COVID-19 pandemic, the group sessions were held virtually, which significantly hindered the opportunities for Ambassadors to engage with their participants. The Ambassadors’ experience in this study may differ from in-person group sessions. Additionally, the composition of participants in the Peers EXCEL program was exclusively female, which may have resulted in gender-dominant conversations. For instance, one of our Peer Ambassadors reported a preference for more same-sex conversations in future programs. Although having more female participants enrolled in health-related programs is common in behavioral interventions, we suggest that future studies aim to balance participants’ demographics when developing recruitment plans. For example, utilizing snowball sampling where male participants reach out to their male peers is a great approach to consider.

## 6. Conclusions

Peer Ambassadors were motivated by their intrinsic beliefs to improve diabetes management in the African American community and reported satisfaction in sharing their experiences and supporting their peers. They valued the educational group sessions for updating their knowledge about diabetes management and sustaining their health-related goals. To sustainably integrate peer support intervention, establishing recognition of milestones for Ambassadors or providing more advanced training to be a group facilitator could be considered in future programs. Time commitment, challenges of interacting with program participants with varied motivation levels, and lack of socialization opportunities were identified as barriers. Future studies should consider these findings by recruiting motivated African American patients to become Ambassadors, offer in-person options for group sessions, and provide training to ensure culturally aware and engaging facilitators.

## Figures and Tables

**Table 1:** Activities of Peer Ambassadors in Peers EXCEL.

	Week	Pre-Intervention			8-Week Intervention								Post-Intervention	
Activities		3	2	1	1	2	3	4	5	6	7	8	10–14	15–24
Peer Ambassador Orientation														
Peer Ambassador Training														
Joint Peer Ambassador and Peer Buddy Meeting														
Group Sessions					Weekly sessions									
Peer Support Phone Calls					Weekly calls								Weekly calls	Biweekly and monthly calls
Peer Ambassador Group Discussions														

Black: Peer Ambassadors only. Gray: Peer Ambassadors and Peer Buddies.

**Table 2:** The facilitator guide for group discussion of Peer Ambassadors’ feedback and experiences of being in the Peers EXCEL program.

Topic	Example Questions
Feedback about 8-week group sessions	Tell me about your experience with the group sessions in Peers EXCEL. In what ways did the program work or not work? Tell me your thoughts about the cultural appropriateness of the Peers EXCEL program? How was the discussion about diet/stress management relevant for African Americans/Blacks? How were the HLWD book and session activities relevant for African American/Blacks?
Feedback about phone calls with Peer Buddies	3. Tell us about what you discussed with your Peer Buddies during the phone calls. 4. Tell us about your experience with the phone calls with your Peer Buddies. What went well with the phone calls? What were some of the challenges? How could they be improved?
Feedback about support from the research team and further training	5. What could be done to improve the support the research team provided to you as Peer Ambassador? What support did you get for connecting and maintaining contact with your PB(s)? 6. What could the study team do to better train and prepare Ambassadors for their role in the program?
Advice about working with future PAs	7. In what ways could Peer Ambassadors’ work with Peer Buddies be improved? 8. What else could the research team do to improve the relationship between peer activities during the program/logistics of the program?
Feedback about Peers EXCEL	9. What was beneficial about serving as a Peer Ambassador for Peers EXCEL? 10. What were the hardest parts about being a Peer Ambassador for Peers EXCEL? How could they be improved?

**Table 3:** Peer Ambassador perceptions and experiences after participating in Peers EXCEL.

Themes	Subthemes	Quotes
Motivation to be a Peer Ambassador	Powerful opportunities to positively impact the African American community	“I have a lot of people in my family that’s been affected by diabetes and… I’m so glad that there’s a program now that’s in place to give us the knowledge and the resources because I didn’t have that. I’m sure none of my family had a chance to experience something like this, an opportunity like that… this is life changing. And that’s the reason why I would be in the program because it is helping people become better. And it’s our people”—Peer Ambassador 3
	Being in a prior similar situation with lack of information and support	“I just remember when I was diagnosed and not having any support, anyone to talk to, not knowing what to do, how to talk to a doctor, how to talk to a pharmacist, what numbers meant. And when you get someone who’s changed because of the information they received, it’s very motivational. … So primarily, it’s just if I can do anything or help anyone with their numbers or understanding all the food and reading labels and exercise. It’s not that I’m 100% correct or anything like that, but I do know how to take my medication”—Peer Ambassador 2
	Paying it forward by sharing one’s experience	“I think that’s big time to be able to have a Peer Buddy. It also just kind of gives them recognition that, hey, you went through a program. You get it. You understand. You were helped. And now your testimony can help somebody else. I think that’s powerful”—Peer Ambassador 3
Program elements that supported Peer Ambassador role	Personal benefits from attending sessions with buddies	“[Attending the group sessions] helps you stay on your game. And it keeps you on top of, and then you get to add to that conversation, your experiences, and let them know that you’re no different. You struggled at it one time. You got to this point. But they get to have a personal relationship with you also”—Peer Ambassador 2
	Session information about goal setting and self-management led to personal commitment	“I think the main topics of diet, exercise, they were so key, and they were so motivational to the Buddies and us. Just like [Peer Ambassador 3] said, we weren’t even required to make commitments. And we all did just because of that energy level”—Peer Ambassador 2 “One thing [the facilitator in HLWD] did was a lot of goal setting. And we didn’t necessarily have to set goals, but it made you want to participate and set some goals for yourself. So, I really did like that, and it keeps me in line with what I need to do with working”—Peer Ambassador 2
	Resources and guides enhanced role preparation	“We have an outline of… when to call and just different topics and things that we should be talking to our Peer Buddies about as well, which is very helpful. Not that we have to follow the script, but at least that gives us a guide, like a map that we should be following just to make sure that we’re hitting those certain touch points”—Peer Ambassador 3 “I’m learning new stuff. When we’re able to have people come on and facilitate and just continue to give us resources, to give the Peer Buddies resources, it not only helps the Peer Buddies, but it helps also the Ambassadors… We get our materials and stuff on time, and so we’re able to kind of be prepared to work with the Buddies and be able to allow them to give feedback”—Peer Ambassador 3
Key elements of achieving engagement	A comfortable and safe environment	“I thought they participated well. They told personal stories. It was like a little group, a little family. You know, everybody all participated. …everybody told their story and felt comfortable to tell their story in that environment. That was really important too. So I thought they [Peer Buddies] were engaged”—Peer Ambassador 2
	Facilitator skill in engaging participants in educational sessions	“It was very huge having her [the facilitator in HLWD] come and kind of do her things and doing check-ins and different things like that, keeping people engaged and, holding people accountable but not being too tough on them and giving people goals week to week… they’re able to kind of take ownership in the things that they decide that they want to do. So I thought that was very awesome… just her energy and personality… it was unbelievable”—Peer Ambassador 3
	Facilitator skill to enhance participant accountability and ownership	“…[the facilitator in HLWD]… holding people accountable but not being too tough on them and giving people goals week to week… they’re able to kind of take ownership in the things that they decide that they want to do”—Peer Ambassador 3
	Discussion of culturally tailored elements aligned with African Americans’ needs	“Having the group of African Americans and talking about their experiences, and then when you go to diet, they’re bringing the food that they like. And it’s something we all like… by having [the facilitator in HLWD], answering questions specifically that we can relate to. Go to a barbecue… Go get the greens, the yams… I may not have gotten that information in another diabetic class, except for we know that we like those things at barbecues”—Peer Ambassador 2
	Introduction to building rapport with Peer Buddies	“I do like the introduction because we have to do it, no matter how awkward… Now that we know the flow of this program, that can even change because we can kind of give them an idea of what to expect and what’s going to happen in the next couple of weeks… I always start off with a text and say, hey, what time can you talk? They get a chance to meet you and see that you… have some of the same struggles they do”—Peer Ambassador 2
	Peer accountability and empowerment	“When you educate people, and they understand how they should be eating, how serious they should take their medication, how they should be engaged and have accountability partners with the group and people that’s checking on them, people that care about them, you know what I’m saying, that’s not even part of their family but just part of a program, to be a part of something, that is huge”—Peer Ambassador 3
Challenges related to being a Peer Ambassador	Hard to maintain individual commitment with long session length and program duration	“I liked the program. But I have to tell you, the sessions were, for me, very long, for two and a half hours, and being committed to that every Thursday… that’s way too much. Now the information is great, and maybe not for the buddies, but for me, it was, six weeks was way too long for me… I think it got hard for people to maintain that schedule for that length of time. And I always thought that was a problem with that particular program. I think people tried very hard…Some was so dedicated because they were getting so much out of it from [the facilitator in HLWD]”—Peer Ambassador 1 “Sometimes it would take three to five phone calls to get to that one conversation, that when we had that conversation, it was great… I just wanted it to be as important to them, you know, that you think about my time too, that it shouldn’t take me four to five phone calls to get to you. That was my biggest challenge”—Peer Ambassador 2
	Easily distracted in a virtual meeting	“Either towards the end or the middle where we get a chance to kind of come together and see each other… I just think that would kind of be motivating. …because a lot of times, when you’re doing so much virtual…, your family, your kids that’s around. So you’re just kind of a little distracted where you’re not in tune as much… if we can do some in person but then do these virtual, I think that’s maybe… good and keep the Peer Buddies motivated”—Peer Ambassador 3
	In-person interactions are more valuable than virtual	“I just think the virtual stuff is just a killer. But if it’s in person, the program is just a lot smoother, more participation. And you can kind of spread it out, you know what I mean, engage, socialize a little bit. So you can pick your points… and people still get the education they need”—Peer Ambassador 3

## Data Availability

Data is available on request.
